# Evaluation of a newly developed rapid ELISA to detect anti-*Ehrlichia canis* antibodies in dogs

**DOI:** 10.1051/parasite/2025054

**Published:** 2025-09-25

**Authors:** Irene Ferrero, Paolo Poletti, Enrica Giachino, Paola Dall’Ara, Joel Filipe

**Affiliations:** 1 Agrolabo S.p.A. 10010 Scarmagno Italy; 2 Department of Veterinary Medicine and Animal Sciences, University of Milan 26900 Lodi Italy

**Keywords:** *Ehrlichia canis*, Ehrlichiosis, EhrlichiaCHECK Ab, ELISA, IFAT

## Abstract

Canine monocytic ehrlichiosis (CME) is a globally prevalent disease with zoonotic potential primarily caused by the obligate intracellular bacterium *Ehrlichia canis*, transmitted by the ticks *Rhipicephalus* spp. to vertebrate hosts. In dogs, CME affects immune system cells, leading to subclinical infection or acute and chronic disease forms that impact multiple organs and potentially result in death if diagnosis is delayed. Diagnosis of canine ehrlichiosis is complex, typically involving cytology, polymerase chain reaction), and serological assays such as the indirect immunofluorescence antibody test (IFAT), considered the gold standard, and enzyme-linked immunosorbent assay (ELISA). Herein, we introduce EhrlichiaCHECK Ab ELISA, a novel rapid indirect ELISA for detecting anti-*E. canis* antibodies in canine serum or plasma samples. The assay’s performance was validated using 112 canine samples (61 negative, 51 positive). Compared to IFAT, the ELISA exhibited high sensitivity (96.1%, 95% confidence interval [CI]: 85.4%–99.3%) and specificity (95.1%, 95% CI: 85.4%–98.7%). Furthermore, in comparison to the widely used commercial INgezim Ehrlichia ELISA (Gold Standard Diagnostics), EhrlichiaCHECK Ab ELISA demonstrated 98.0% agreement and enhanced specificity attributed to a more specific antigen, corroborated by bioinformatics analysis. The assay also demonstrated accuracy, with low percentage values of intra- and inter-assay coefficients of variation. In conclusion, our data suggest that the newly developed assay is a valuable tool for diagnosing *E. canis* infections in dogs.

## Abstract (fr)

L’ehrlichiose monocytaire canine (EMC) est une maladie mondialement répandue à potentiel zoonotique, principalement causée par la bactérie intracellulaire obligatoire *Ehrlichia canis*, transmise par les tiques *Rhipicephalus* spp. aux hôtes vertébrés. Chez le chien, l’EMC affecte les cellules du système immunitaire, entraînant une infection subclinique ou des formes aiguës et chroniques de la maladie affectant plusieurs organes et potentiellement mortelles en cas de diagnostic tardif. Le diagnostic de l’ehrlichiose canine est complexe et implique généralement la cytologie, la réaction en chaîne par polymérase (PCR) et des tests sérologiques tels que le test d’immunofluorescence indirecte (IFAT), considéré comme la référence, et le test d’immuno-absorption enzymatique (ELISA). Nous présentons ici EhrlichiaCHECK Ab ELISA, un nouveau test ELISA indirect rapide pour la détection des anticorps anti-*E. canis* dans des échantillons de sérum ou de plasma canin. Les performances du test ont été validées à l’aide de 112 échantillons canins (61 négatifs, 51 positifs). Comparé à l’IFAT, l’ELISA a montré une sensibilité (96,1 %, intervalle de confiance à 95 % [IC] : 85,4 %–99,3 %) et une spécificité (95,1 %, IC à 95 % : 5,4 %–98,7 %) élevées. De plus, par rapport au test ELISA commercial INgezim Ehrlichia largement utilisé (Gold Standard Diagnostics), l’ELISA EhrlichiaCHECK Ab a démontré une concordance de 98,0 % et une spécificité améliorée attribuée à un antigène plus spécifique, corroboré par une analyse bioinformatique. Le test a également fait preuve de précision, avec de faibles valeurs en pourcentage des coefficients de variation intra- et inter-tests. En conclusion, nos données suggèrent que le test nouvellement développé est un outil précieux pour le diagnostic des infections à *E. canis* chez les chiens.

## Introduction

Canine monocytic ehrlichiosis (CME) is a vector-borne zoonotic disease with a global distribution. The primary vector is the tick *Rhipicephalus sanguineus* s.l., while mammals, including dogs, cats, and humans, serve as vertebrate hosts [7, 29, 56]. CME is mainly caused by *Ehrlichia canis*, although *Ehrlichia chaffeensis* can occasionally be involved. Both species are capable of infecting dogs and humans, leading to severe disease manifestations [9, 56]. *Ehrlichia canis* is an obligate intracellular Gram-negative bacterium that, after the tick takes a blood meal, localizes in the midgut and then the salivary glands of the tick. During subsequent feeding, it is transmitted to a healthy dog through the tick’s saliva. Once in the host, *E. canis* targets immune system cells, particularly macrophages, lymphocytes, and monocytes. Inside the cytoplasm, the bacterium differentiates and multiplies through binary fission within the phagosome, forming endosomes that take on a structure known as a “morula,” which contains numerous *E. canis* bacteria. Eventually, *E. canis* is released via exocytosis or cell lysis, allowing the infection to spread further [4].

After an incubation period of 8–20 days, depending on the *Ehrlichia* spp., ehrlichiosis manifests in acute, subclinical, and chronic forms that affect multiple organs and systems, including the lungs, meninges, and kidneys, and can lead to death if not diagnosed in time [4]. The acute phase typically lasts 2–4 weeks and is characterized by high fever, depression, lethargy, anorexia, lymphadenomegaly, splenomegaly, and hemorrhagic tendencies that may vanish even without chemotherapeutic treatment. In some cases, the animal may maintain the disease in a subclinical form, thus becoming an important source of infection without presenting any clinical manifestation for an extended period of months or years. During this phase, the animal appears clinically normal, with no evident signs of illness, although mild thrombocytopenia can be detected through hematological testing. Some subclinically infected dogs may progress to the chronic stage, which is the most severe and fatal form of the disease, characterized by myelosuppression and severe pancytopenia. Clinical signs are similar to those in the acute phase but more severe [4, 26]. Previous studies have identified a small group of major immunoreactive proteins in *E. canis*, including tandem repeat proteins (TRPs), an ankyrin repeat protein (Ank200), and the major outer membrane protein (OMP) family, which stimulate the antibody response during infection in both humans and dogs. Among the TRPs, TRP19 (or gp19), TRP36 (or gp36), TRP95, and TRP140 are all significant immunoreactive proteins [37].

The diagnosis of CME is complex. In acute cases, cytology can provide an earlier diagnosis through light microscopy by detecting typical cytoplasmic *E. canis* morulae in monocytes on blood smears. However, this method is considered labor-intensive and has low diagnostic sensitivity during the subclinical and chronic phases [4]. On the other hand, serological methods are valuable screening and diagnostic tools. Among these, there are the indirect immunofluorescence antibody test (IFAT), considered the gold standard, and the enzyme-linked immunosorbent assay (ELISA). However, a definitive diagnosis requires molecular techniques, such as DNA amplification by polymerase chain reaction (PCR) [26, 61]. CME can be fatal if not treated promptly and requires rapid and accurate diagnosis to initiate the most appropriate pharmacological treatment. To address this need, the R&D team of veterinary diagnostic kit supplier Agrolabo S.p.A. has developed a new ELISA test, called EhrlichiaCHECK Ab ELISA, introduced in this paper. In addition to being a rapid and qualitative assay for the detection of anti-*E. canis* antibodies in canine samples, it is sensitive and more specific than the previous version and commercialised tests because it is based on a novel antigen that limits cross-reactivities towards other *Ehrlichia* species and, therefore, reduces false-positive case detection.

## Materials and methods

### Ethics statement

This study did not involve human participants. Canine serum or plasma samples were collected with owner consent during routine veterinary evaluations and voluntarily donated for research purposes. The procedures performed are not considered animal experimentation and therefore are not subject to the Italian Legislative Decree 26/2014 and Directive 2019/63/EU of the European Parliament.

### Samples collected in the study

A total of 112 serum or plasma samples were collected from four veterinary clinics from north and central Italy (Piedmont and Lazio regions), as well as from Greece, primarily from the Attica region. These samples were tested for anti-*Ehrlichia* antibodies using the gold standard method, IFAT, and were classified as negative or positive (61 negative and 51 positive), then used for all stages of test development. To ensure comprehensive representation and broad applicability of the test, no sample selection was performed. Consequently, information regarding the samples or the patients’ clinical condition was unknown. High lipemic or hemolytic samples were also included in the study. The number of samples tested is consistent with other bibliographic studies involving ELISA testing [2, 41, 52, 66].

### Plate preparation and EhrlichiaCHECK Ab ELISA

Nunc PolySorp microplates (Thermo Fisher Scientific, Waltham, MA, USA) were used for test development, as they are commonly employed to immobilize both antigens [19, 55] and antibodies [49] in ELISA testing. To prepare the plates, we followed internal company protocols as well as standardized procedures [6, 17]. The microplates were coated with recombinant antigen derived from the *E. canis* glycoprotein gp19 (Rekom Biotech, Granada, Spain) at two concentrations, 0.5 μg/mL and 1 μg/mL, diluted in carbonate-bicarbonate (CB) buffer (pH 9.6) (Agrolabo S.p.A., Scarmagno, Italy), as previously described [17, 23]. In the final protocol, microplates were coated with 0.5 μg/mL of *E. canis* antigen and incubated at +2–8 °C overnight (ON). The plates were then washed, blocked with a commercial blocking solution (Surmodics, Eden Prairie, MN, USA) for 1 h at room temperature (RT), and allowed to dry at RT for 24 h before being stored at +2–8 °C.

During ELISA setup, four types of positive controls (PCs) were used, including three anti-histidine (His) tag antibodies labeled with horseradish peroxidase (HRP) of different suppliers (PC1 from Sigma-Aldrich, St. Louis, MO, USA; PC2 from Abcam Limited, Cambridge, UK; PC3 from Thermo Fisher Scientific) and one formed by a pool of canine positive sera (PC4). The negative control (NC) and samples, diluted 1:100 in a diluent formed by phosphate-buffered saline (PBS) and bovine serum albumin (BSA) (Agrolabo S.p.A.), were added in 100 μL volume in each well, followed by a 15-minute incubation at RT. After washing to remove unbound specimens, 100 μL of the conjugate anti-dog IgG antibody labeled with HRP (Bethyl Laboratories Inc., Montgomery, TX, USA) were added, followed by a 15-minute incubation at RT. After a second wash cycle and the addition of 100 μL of substrate/chromogen 3,3’,5,5’-Tetramethylbenzidine (TMB) (Surmodics), the plates were incubated for 5 min at RT in the dark producing a colorimetric reaction (blue indicating positive samples, no color indicating negative ones). The reaction was stopped by adding 100 μL of stop solution (0.2 M sulfuric acid from Agrolabo S.p.A.) and results were interpreted instrumentally by measuring the optical density (OD) at 450 nm using an ELISA microplate reader (Multiskan SkyHigh, Thermo Fisher Scientific). The test was considered valid if the OD value for the PC was above 0.6, and the OD for the NC was below 0.1.

### ELISA validation

Since IFAT is generally considered the serological “gold standard” test for detecting exposure to *E. canis* in dogs [26, 46, 51, 61], EhrlichiaCHECK Ab ELISA was validated by testing 112 canine sera (51 positives and 61 negatives) against the IFAT FLUO *Ehrlichia canis* kit (Agrolabo S.p.A.), following the manufacturer’s instructions. The IFAT kit consisted of slides coated with canine macrophages infected with *E. canis* which were observed under a fluorescence microscope equipped with an FITC filter. Samples in which the cells showed no yellow-green fluorescence or appeared greyish-red were classified as negative, while those with bright yellow-green fluorescence and stained inclusion bodies (morulae) in the cytoplasm were considered positive. A sample was considered positive if the first dilution (1:40) showed a positive result.

### Comparative analysis

The performance of EhrlichiaCHECK Ab ELISA was assessed by analyzing 51 canine samples (24 positives and 27 negatives) and comparing the results with those from the widely used INgezim Ehrlichia ELISA (Gold Standard Diagnostics, Madrid, Spain) [16, 32]. In all cases, IFAT was considered the reference method. The INgezim Ehrlichia ELISA was performed according to the manufacturer’s instructions.

### ELISA accuracy assessment

Precision and accuracy were evaluated by calculating the intra- or inter-assay coefficients of variation (CV), which were obtained within a single run or across different runs, respectively. Positive and negative samples were tested by two operators on different days and at various times, as described in the guidelines [63], with 8 replicates on 3 separate batches for a total of 24 repetitions per sample, in accordance with the International Council for Harmonization (ICH) Q2 (R1) guidance [22]. Test precision was assessed for each sample by calculating the mean, minimum, and maximum optical density (OD) values, standard deviation, and %CV.

### ELISA kit shelf-life evaluation

As there are no specific regulations for stability testing of veterinary diagnostic kits, we referred to the Standard Guide for Accelerated Aging of Sterile Medical Device Packages ASTM F1980-21 [3], which applies the Arrhenius equation to estimate the degradation of medical devices under accelerated conditions. The duration of accelerated storage was determined using online calculators [30, 45], corresponding to 6 weeks at +37 °C, which is equivalent to 18 months of real-time stability at +2–8 °C. For both the accelerated and real-time stabilities, the initial test was performed at time zero (T_0_), followed by assays every 2 weeks for the first 6 weeks. After this period, only kits stored refrigerated at +2–8 °C (real-time conditions) were tested every 3 months up to 18 months. For each sample and control, the percentage of remaining activity (%RA) was calculated as the ratio of the OD value obtained at a specific time to that at time T_0_, with 70% being the lower limit of acceptability [34]. The number of samples tested was consistent with that used in the literature [41, 66].

### ELISA cut-off determination

To enhance the precision of the test, several methods were employed to estimate the ELISA cut-off. In preliminary assays, the cut-off was calculated as the mean OD value of negative samples plus three times the standard deviation (SD) [13, 31, 33]. Samples were classified based on their OD values, 10% above or below the cut-off, as in other commercial ELISA tests [1, 20]. This method and other approaches were used to estimate the cut-off considering 112 samples (51 positive and 61 negative), already utilized in ELISA validation. According to these, the optimal cut-off should correspond to the OD value where the difference between sensitivity and specificity was minimized [38], or as a value between the maximum OD value of negative samples and the minimum OD value of positive samples [33]. Additionally, the optimal cut-off that maximized the difference between true positives and false-positives was estimated using the Receiver Operating Characteristics (ROC) curve and Youden’s index (J), which ranges from 0 (completely ineffective test) to 1.0 (perfectly effective test). Furthermore, the numbers of true positives (a), false-positives (b), false-negatives (c), and true negatives (d) from the comparison of EhrlichiaCHECK Ab ELISA with IFAT were used to calculate diagnostic parameters including accuracy, sensitivity, specificity, positive likelihood ratio (LR+) and negative likelihood ratio (LR−). The 95% confidence interval (CI) was calculated for sensitivity and specificity.

### Bioinformatics analysis

Cross-reactions were theoretically investigated through bioinformatics analysis of the two different antigens used in EhrlichiaCHECK Ab ELISA (Agrolabo S.p.A.) and INgezim Ehrlichia ELISA (Gold Standard Diagnostics). The protein sequences were obtained from the National Center for Biotechnology Information (NCBI) database (*E. canis* p30, GenBank accession number: AAC68667.1; *E. canis* gp19, GenBank accession number: ABU44523.1) and compared using the Basic Local Alignment Search Tool (BLAST) of NCBI.

### Statistical analysis

Statistical analysis was performed for the comparison study using a *t*-test, with a *p*-value < 0.05 considered significant. Cohen’s *d* index was used to measure the strength of the relationship (Effect size: *d* = 0.2 for small effect, *d* = 0.5 for moderate effect, *d* = 0.8 for large effect), and Cohen’s Kappa coefficient index was applied to calculate the degree of agreement beyond chance.

## Results

### Preliminary setup and conjugate dilution selection

Preliminary assays were conducted on microplates coated with 0.5 μg/mL or 1 μg/mL of *E. canis* gp19 antigen by testing 16 samples (12 negative and 4 positive) with conjugate antibody dilutions 1:30,000 and 1:40,000. ELISA accurately distinguished negative from positive samples, with OD values falling below or above the cut-off accordingly. Additionally, no significant differences in OD values were observed between the two coating conditions tested with both conjugates (Table 1).

**Table 1:** Preliminary ELISA test.

Samples	IFAT	Conjugate 1:30,000		Conjugate 1:40,000	
		0.5 μg/mL	1 μg/mL	0.5 μg/mL	1 μg/mL
Sample 1	NEG	n.d.	n.d.	0.129 ± 0.001	0.151 ± 0.004
Sample 2	NEG	n.d.	n.d.	0.056 ± 0.001	0.059 ± 0.001
Sample 3	NEG	n.d.	n.d.	0.082 ± 0.005	0.094 ± 0.004
Sample 4	NEG	n.d.	n.d.	0.050 ± 0.001	0.049 ± 0.000
Sample 5	NEG	0.057 ± 0.001	0.054 ± 0.000	0.056 ± 0.001	0.056 ± 0.002
Sample 6	NEG	0.076 ± 0.004	0.074 ± 0.005	0.074 ± 0.001	0.068 ± 0.002
Sample 7	NEG	n.d.	n.d.	0.070 ± 0.001	0.069 ± 0.000
Sample 8	NEG	n.d.	n.d.	0.054 ± 0.001	0.052 ± 0.003
Sample 9	NEG	n.d.	n.d.	0.057 ± 0.000	0.057 ± 0.003
Sample 10	NEG	n.d.	n.d.	0.049 ± 0.003	0.057 ± 0.001
Sample 11	NEG	n.d.	n.d.	0.054 ± 0.000	0.059 ± 0.000
Sample 12	NEG	n.d.	n.d.	0.049 ± 0.001	0.047 ± 0.002
Sample 13	POS	2.604 ± 0.050	2.533 ± 0.039	2.189 ± 0.025	2.332 ± 0.012
Sample 14	POS	1.985 ± 0.023	1.843 ± 0.003	1.473 ± 0.006	1.554 ± 0.016
Sample 15	POS	2.143 ± 0.016	2.215 ± 0.018	1.761 ± 0.018	1.793 ± 0.005
Sample 16	POS	0.635 ± 0.000	0.638 ± 0.013	0.566 ± 0.040	0.657 ± 0.004
Cut-off		0.175	0.171	0.169	0.173

Plates were coated with 0.5 μg/mL or 1 μg/mL of *E. canis* gp19 antigen. Results are reported as mean OD values ± standard deviation. Each sample was tested diluted 1:100 in duplicate, with the conjugate antibody at 1:30,000 and 1:40,000 dilutions. The cut-off was calculated as the mean OD value of negative samples plus 3 times standard deviation, considering positive or negative samples with OD values 10% above or below the cut-off, respectively. NEG: negative samples; POS: positive sample; n.d.: not determined.

Tests with a longer sample incubation time of 30 minutes performed well and showed no substantial differences in OD values between plates coated with 0.5 μg/mL or 1 μg/mL of *E. canis* gp19 antigen (Supplementary Table S1). Based on these results, for future experiments, we decided to perform ELISA with a 15-minute sample incubation time on plates coated with 0.5 μg/mL *E. canis* antigen and using the 1:30,000 conjugate antibody dilution to achieve optimal performance in a faster and more cost-effective manner.

### Positive and negative control selection

Since no commercially available antibodies were directed against the plate-immobilized antigen, and the recombinant gp19 antigen contains a histidine tag, anti-histidine antibodies labelled with HRP were tested as positive control (PC). During assay development, three types of anti-His antibodies (PC1, PC2, PC3) and a pool of canine positive sera (PC4) were considered. It is known that a strong positive sample should have an OD value of 1.5 or higher [2, 64], with a minimum OD value of 0.6 for test acceptability, as reported in the literature [2, 64] and in other ELISA tests [50, 57, 59]. Anti-His antibodies (PC1, PC2, and PC3) were tested at dilutions of 1:1000 and 1:8000, but all yielded invalid results, with OD values below the acceptable limit of 0.6. However, the pool of positive canine sera (PC4), tested at dilutions of 1:100, 1:150, and 1:200, yielded good results, with OD values above 0.6 (Table 2).

**Table 2:** ELISA positive and negative controls evaluation.

Samples	Dilution	Mean OD ± SD
PC1	1:1000	0.089 ± 0.000 (*n* = 2)
PC2	1:1000	0.105 ± 0.006 (*n* = 2)
	1:8000	0.058 ± 0.001 (*n* = 2)
PC3	1:1000	0.127 ± 0.002 (*n* = 2)
	1:8000	0.069 ± 0.002 (*n* = 2)
PC4	1:100	1.680 ± 0.088 (*n* = 16)
	1:150	1.426 ± 0.062 (*n* = 16)
	1:200	1.279 ± 0.060 (*n* = 16)
Negative samples	1:100	0.083 ± 0.040 (*n* = 58)
NC	–	0.051 ± 0.004 (*n* = 10)

Three anti-His tag HRP antibodies (PC1, PC2, and PC3) were tested at dilutions 1:1000 and 1:8000. ELISA results are reported as mean OD ± standard deviation (SD). PC1: monoclonal anti-His HRP antibody (Sigma-Aldrich); PC2: polyclonal anti-His tag HRP antibody (Abcam Limited); PC3: monoclonal anti-His tag HRP antibody (Thermo Fisher Scientific); PC4: pool of canine positive sera (1:100, 1:150, 1:200); NC: negative control (sample diluent); n: number of tests.

For the negative control (NC), we tested a sample diluent composed of PBS and BSA, as used in other ELISA tests [21, 25, 28, 43, 48]. This NC was tested in 10 replicates, and all OD values were below 0.1, within the acceptable OD limit of 0.3 [2, 64] (Table 2). Additionally, we tested 58 negative samples diluted 1:100 to ensure their signal was similar to that of the new NC. In this case, the results were also satisfactory, with OD values below 0.1 and the mean OD value within the range defined by the standard deviation of the negative samples, which supports the use of the sample diluent as the NC.

### Estimation of ELISA cut-off value

**Figure 1:**
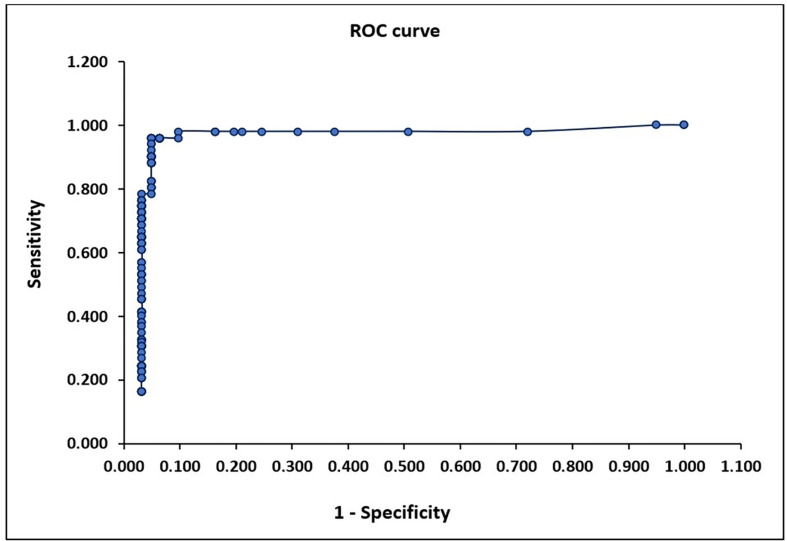
Cut-off determination by ROC curve. The graph visualizes the diagnostic ability of a test by plotting the true positive rate (sensitivity) against the false positive rate (1 – specificity) at various threshold settings. The upper left point in the curve corresponds to the best cut-off. In our case, sensitivity was 0.961 (96.1%) and the false-positive rate was 0.049 (low probability, 4.9%).

**Figure 2:**
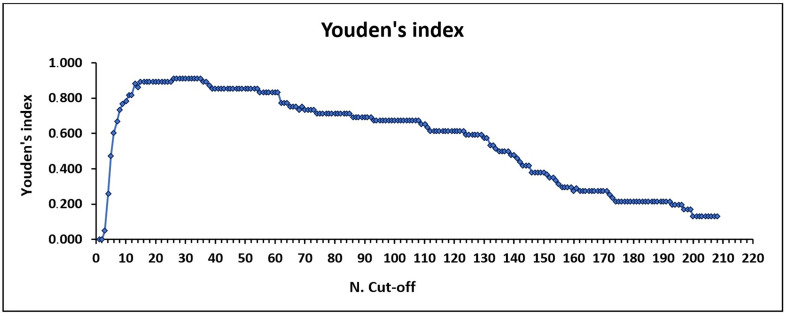
Cut-off determination by Youden’s index. The maximum value of Youden’s index represented the best test cut-off value and corresponded to 0.912. This value is maintained from cut-off number (*N*. Cut-off) 26 to 35.

The detailed results obtained for the 112 samples (51 IFAT-positive and 61 IFAT-negative) used for the cut-off estimation and kit validation are described in Supplementary Table S2. The preliminary cut-off was confirmed using several methods. According to literature reports, the cut-off for the new ELISA should fall between the maximum OD value of negative samples (OD 0.275) and the minimum OD value of positive samples (OD 0.375) [33], or around an OD value of 0.280, representing the point where the difference between sensitivity and specificity is minimal (difference of 0.010) [38]. At the OD cut-off of 0.280, sensitivity and specificity were 96.1% and 95.1%, respectively. These parameters remained stable up to an OD cut-off of 0.370 (Supplementary Table S3). Finally, both the ROC curve (Figure 1) and Youden’s index indicated that the optimal cut-off was at an OD value of 0.280 (maximum J value: 0.912) (Figure 2). Although the precise cut-off for EhrlichiaCHECK Ab ELISA was OD 0.280, since sensitivity and specificity remained unchanged up to OD 0.370, we selected an OD value of 0.300 as the final cut-off for the kit, with a 10% uncertainty level corresponding to the “doubtful zone”.

### EhrlichiaCHECK Ab ELISA validation

**Figure 3:**
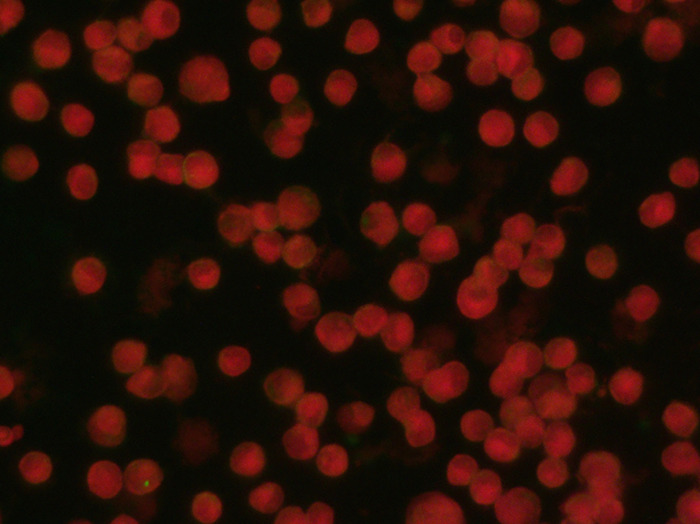
Example of IFAT-negative result obtained on a negative sample observed at 100× by using the IFAT kit FLUO *Ehrlichia canis* (Agrolabo S.p.A.).

**Figure 4:**
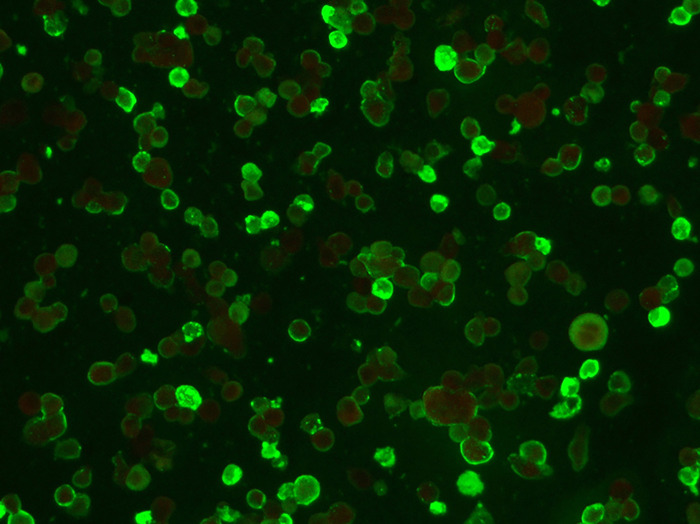
Example of IFAT-positive result obtained on a positive sample observed at 100× by using the IFAT kit FLUO *Ehrlichia canis* (Agrolabo S.p.A.).

The EhrlichiaCHECK Ab ELISA was validated against the IFAT, which was used as the reference method, utilizing the same samples previously used for cut-off determination (Supplementary Table S2). Figure 3 and Figure 4 show examples of both negative and positive results, respectively, obtained using IFAT. The ELISA cut-off was calculated as described earlier and set at OD 0.300. The new assay identified 52 positives (compared to 51 by IFAT) and 60 negatives (compared to 61 by IFAT), resulting in three false-positives and two false-negatives (Table 3, Supplementary Table S2). Compared to IFAT, the EhrlichiaCHECK Ab ELISA demonstrated 95.5% agreement, with sensitivity of 96.1% (95% CI: 85.4%–99.3%) and specificity of 95.1% (95% CI: 85.4%–98.7%). Cohen’s Kappa coefficient was 0.904, indicating very good agreement. The test accuracy was 100%, the negative likelihood ratio (LR−) was 0.041, and the positive likelihood ratio (LR+) was 19.536.

**Table 3:** Validation results of EhrlichiaCHECK Ab ELISA against IFAT (reference test).

EhrlichiaCHECK Ab ELISA (Agrolabo S.p.A.)	IFAT (FLUO *Ehrlichia canis*, Agrolabo S.p.A.) – Reference test		
	Positive	Negative	Total
Positive	49^(a)^	3^(b)^	52
Negative	2^(c)^	58^(d)^	60
Total	51	61	112

Total number of samples analyzed: 112. (a) True positives; (b) false positives; (c) false negatives; (d) true negatives.

### Reproducibility and precision assessment

The accuracy of the EhrlichiaCHECK Ab ELISA was evaluated by testing controls, two positive samples, and two negative samples in 8 replicates across 3 batches, totaling 24 repetitions for each sample. Detailed results are provided in Supplementary Table S4. The EhrlichiaCHECK Ab ELISA exhibited high accuracy, with both intra- and inter-assay coefficients of variation (%CV) remaining within the acceptable 20% limit [15] (Table 4).

**Table 4:** Reproducibility and intra- and inter-assay coefficients of variation.

Batches	Parameters	PC	NC	Sample 1 (−)	Sample 2 (−)	Sample 3 (+)	Sample 4 (+)
Batch 1	Mean OD ± SD	2.186 ± 0.055	0.046 ± 0.002	0.057 ± 0.003	0.065 ± 0.003	2.211 ± 0.053	2.138 ± 0.043
	Minimum OD	2.108	0.044	0.054	0.060	2.136	2.072
	Maximum OD	2.280	0.049	0.061	0.069	2.264	2.202
	Intra-assay %CV	2.498	4.374	4.437	4.817	2.419	1.997
Batch 2	Mean OD	2.023 ± 0.038	0.051 ± 0.003	0.056 ± 0.003	0.061 ± 0.003	2.000 ± 0.065	2.077 ± 0.044
	Minimum OD	1.983	0.047	0.052	0.057	1.900	1.988
	Maximum OD	2.072	0.055	0.062	0.064	2.096	2.126
	Intra-assay %CV	1.865	5.377	5.570	5.075	3.252	2.097
Batch 3	Mean OD	2.084 ± 0.083	0.048 ± 0.003	0.056 ± 0.003	0.062 ± 0.003	2.083 ± 0.081	2.161 ± 0.079
	Minimum OD	1.988	0.044	0.054	0.056	1.934	2.003
	Maximum OD	2.206	0.053	0.062	0.066	2.166	2.262
	Intra-assay %CV	3.971	6.106	5.524	5.049	3.906	3.675
Inter-assay %CV		2.778	5.286	5.177	4.980	3.192	2.590

ELISA tests were performed in 8 replicates on 3 plate batches (24 tests for each sample). PC: positive control; NC: negative control. Samples: negatives (*n* = 1, *n* = 2), positives (*n* = 3, *n* = 4). OD: optical density; SD: standard deviation. CV: coefficient of variation.

### Comparison study with commercial kit

The performance of the EhrlichiaCHECK Ab ELISA was compared to the commercially available INgezim Ehrlichia ELISA (Gold Standard Diagnostics) by analyzing 51 canine samples (24 positives and 27 negatives). The new test showed agreement of 98.0% with the INgezim Ehrlichia ELISA (Cohen’s Kappa: 0.961, indicating very good agreement) (Table 5). Overall, we obtained good results for both positive and negative samples, with one false-positive observed in the INgezim Ehrlichia ELISA (sample *n =* 11, OD value 2.604) (Supplementary Table S5). Table 6 shows a comparison of both ELISA methods against IFAT (reference method). Agreement with the first assay was 100% (100% sensitivity, 95% CI: 82.8%–100%; 100% specificity, 95% CI: 84.5%–100%; Cohen’s Kappa: 1.000), while the agreement with the second assay was 98.0% (100% sensitivity, 95% CI: 82.8%–100%; 96.3% specificity, 95% CI: 79.1%–99.8%; Cohen’s Kappa: 0.961). Thus, the EhrlichiaCHECK Ab ELISA demonstrated higher specificity compared to the INgezim Ehrlichia ELISA.

**Table 5:** Comparison results of EhrlichiaCHECK Ab ELISA (Agrolabo) against the INgezim Ehrlichia ELISA (Gold Standard Diagnostics).

INgezim Ehrlichia ELISA (GSD)	EhrlichiaCHECK Ab ELISA (Agrolabo S.p.A.)		
	Positive	Negative	Total
Positive	24^(a)^	1^(b)^	25
Negative	0^(c)^	26^(d)^	26
Total	24	37	51

The Agrolabo’s kit was taken as reference method. Number of samples analyzed: 51. GSD: Gold Standard Diagnostics. (a) True positives; (b) false positives; (c) false negatives; (d) true negatives. Proportion of agreement (a + d/a + b + c + d): 98.0%; Cohen’s Kappa: 0.961.

**Table 6:** Comparison results of EhrlichiaCHECK Ab ELISA (Agrolabo) and INgezim Ehrlichia ELISA (Gold Standard Diagnostics) against IFAT.

Assays	Samples	IFAT		
		Positive	Negative	Total
EhrlichiaCHECK Ab ELISA (Agrolabo)	Positive	24^(a)^	0^(b)^	24
	Negative	0^(c)^	27^(d)^	27
	Total	24	27	51
INgezim Ehrlichia ELISA (GSD)	Positive	24^(a)^	1^(b)^	25
	Negative	0^(c)^	26^(d)^	26
	Total	24	27	51

The IFAT (FLUO *Ehrlichia canis* kit from Agrolabo S.p.A.) was considered the reference method. Total number of samples analyzed: 51. GSD: Gold Standard Diagnostics. (a) true positives; (b) false positives; (c) false negatives; (d) true negatives. Proportion of agreement (a + d/a + b + c + d): 100% (EhrlichiaCHECK Ab ELISA) and 98.0% (INgezim Ehrlichia ELISA).

Additionally, statistical analysis using a *t*-test (*p*-value < 0.05) comparing the mean OD values of the two assays revealed that both the POSITIVE and negative OD values in the EhrlichiaCHECK Ab ELISA were statistically significantly lower than those in the INgezim Ehrlichia ELISA. However, the effect sizes, calculated using Cohen’s *d* index, were 1.113 for negative samples (indicating a large difference) and 0.080 for positive samples (indicating a small difference) (data not shown).

### Cross-reaction evaluation by bioinformatics analysis

It is known that the INgezim Ehrlichia ELISA uses the recombinant p30 protein of *E. canis* [36], while our EhrlichiaCHECK Ab ELISA employs the *E. canis* gp19 antigen. Since *Ehrlichia* spp. share similar antigenic molecules [61], we performed a bioinformatics analysis to theoretically assess potential cross-reactions, which may explain the differences in sensitivity and specificity observed between the two ELISA kits. We applied the commonly accepted criterion that two sequences are homologous if they are more than 30% identical over their entire lengths [47].

From the analysis of the *E. canis* p30 protein, we found that it shares significant similarity with many other *Ehrlichia* spp. antigens. Specifically, high similarity (greater than 60%) was observed with the P44/Msp2 family outer membrane protein (OMP) in *E. canis*, *Ehrlichia minasensis*, *Ehrlichia muris*, *Ehrlichia japonica*, *E. chaffeensis*, and *Ehrlichia ruminantium*; the surface antigen Msp4 of *E. minasensis*; the P28 antigen of *E. chaffeensis*, *E. canis*, and *E. muris*; OMP-1 of *Ehrlichia ewingii*; p30-1 and p30-2 of *E. canis*; and Major Antigenic Protein 1 (Map1) of *E. ruminantium* (Table 7). In contrast, we found that the *E. canis* gp19 antigen shares similarity with the P13 protein of *E. muris* (greater than 40%) and with the “hypothetical protein” of *E. minasensis* (greater than 50%), *E. muris*, and *E. japonica* (greater than 40%) (Table 7). These “hypothetical proteins” are now identified as TRP proteins, including TRP19 (or gp19) [37].

**Table 7:** Similarity research through bioinformatics analysis on *E. canis* p30 and gp19 antigens.

Antigen investigated	Protein	Microrganism	Percent identity
p30	P44/Msp2 family outer membrane protein	*Ehrlichia canis*	67.7%–100%
		*Ehrlichia chaffeensis*	64.8%–71.5%
		*Ehrlichia japonica*	69.1%–72.9%
		*Ehrlichia minasensis*	66.7%–86.9%
		*Ehrlichia muris*	69.4%–73.6%
		*Ehrlichia ruminantium*	62.1%–63.0%
	Surface antigen msp4	*Ehrlichia minasensis*	63.0%–84.1%
	28kDa outer membrane protein (p28)	*Ehrlichia canis*	67.7%–68.0%
		*Ehrlichia chaffeensis*	64.4%–71.5%
		*Ehrlichia muris AS145*	70.1%–70.5%
	Outer Menbrane Protein 1 (OMP-1)	*Ehrlichia ewingii*	64.0%
	Major outer membrane protein p30-1	*Ehrlichia canis*	67.7%
	Major outer membrane protein p30-2	*Ehrlichia canis*	68.8%
	Major antigenic protein 1 (Map1)	*Ehrlichia ruminantium*	63.1%
		*Ehrlichia ruminantium str. Gardel*	62.8%
TRP19 (gp19)	Outer Menbrane Protein P13	*Ehrlichia muris subsp. eauclairensis*	62.5%
		*Ehrlichia muris AS145*	46.5%
	Hypothetical protein	*Ehrlichia japonica*	43.3%
		*Ehrlichia minasensis*	51.4%
		*Ehrlichia muris*	43.9%–62.5%

The sequences of *E. canis* p30 (GenBank accession number: AAC68667.1) and *E. canis* gp19 (GenBank accession number: ABU44523.1) were compared to all proteins of *Ehrlichia* spp. by NCBI-BLAST.

### Estimation of ELISA test expiry

During all analytical sessions conducted in the accelerated stability study at +37 °C for 6 weeks, both samples and controls showed a percentage of remaining activity (% RA) consistently above 70% (Supplementary Table S6). These results were further confirmed in the long-term stability study (data not shown).

## Discussion

CME is the most severe form of canine ehrlichiosis and is found worldwide. Three major immunoreactive tandem repeat proteins (TRPs) including TRP19, TRP36, and TRP140 have been identified as key markers for diagnosing *E. canis* infection. Among these, the highly conserved protein TRP19 (also known as gp19) is the most reliable and preferred for the immunodiagnosis of CME [37, 46]. In this study, we present the new EhrlichiaCHECK Ab ELISA, which is based on the gp19 antigen of *E. canis* and developed with IFAT as the gold standard method [26, 46, 51, 61]. The new ELISA demonstrated excellent agreement with IFAT, showing 95.5% concordance, with sensitivity of 96.1% (95% CI: 85.4%–99.3%) and specificity of 95.1% (95% CI: 85.4%–98.7%). It can be conducted at RT without the need for specialized laboratory equipment, requiring only an ELISA microplate reader for result interpretation. Furthermore, the assay exhibited high accuracy, with both intra- and inter-assay %CVs within the acceptable limits [15, 34].

Since an anti-gp19 antibody was not commercially available at the time of test development, we explored the use of anti-histidine antibodies as positive controls (PCs) as an alternative to canine samples. It is well-known that histidine tags are commonly used as affinity tags for purifying recombinant proteins [8] and for their detection by antibodies [18, 27, 35, 65]. Unfortunately, the three anti-His antibodies tested produced invalid results, with OD values below the acceptable limit of 0.6. In contrast, only the pool of canine positive sera proved to be an effective PC. We hypothesize that during the coating process, the antigen likely folds in a way that prevents the exposure of the histidine tail, thus not allowing recognition by the anti-histidine antibodies. Unlike the PC, which worked only with positive samples, the NC formed by sample diluent, rather than negative specimens, proved valuable. The mean OD of the NC was consistently below 0.1, well within the OD limit of 0.3 and aligned with the values obtained from negative samples. Notably, PBS-based solutions are already used as NCs in ELISA tests designed for both scientific purposes and commercially available assays [21, 25, 28, 43, 48].

We demonstrated the absence of non-specific binding to the coating constituents by analyzing both controls and samples. However, potential interference from substances in the blood, such as pharmaceuticals or naturally occurring compounds linked to inflammation, should be considered. We have not conducted in-depth studies to determine cross-reactivity reactions, in part due to the challenges in obtaining representative samples.

A positive result from ELISA or IFAT only indicates past or present infection and does not necessarily reflect the current disease status [4]. During the ELISA validation process, three false-positive and two false-negative results were detected. Since *Ehrlichia* spp., particularly those within the same genogroup, share similar antigenic molecules, serological tests may be affected by cross-reactions. It is known that IFAT can be influenced by cross-reactivity between *E. canis* and *E. chaffeensis* due to shared OMP proteins such as p30, p30-1, and p30a [44, 61]. As partially confirmed by our bioinformatics analysis, these proteins exhibit significant similarity to those in the OMP-1 gene family of *E. chaffeensis* [44] and the major antigen protein 1 (MAP-1) of *E. ruminantium* [60]. Additionally, *E. muris* may cross-react serologically with *E. chaffeensis* and *E. canis* [62]. Notably, a cross-reaction has also been observed between the p30 antigen of *E. canis* and seropositive *Leishmania infantum* samples [36]. Moreover, canine sera from *E. ewingii*, known to induce the granulocytic ehrlichiosis in dogs and tested against *E. canis* antigens showed binding patterns similar to those of anti-*E. canis* sera [54, 61]. Cross-reactions have also been documented between *E. canis* and species within the *E. phagocytophila* genogroup, as well as with *Neorickettsia helminthoeca*, the causative agent of salmon poisoning disease [53, 61]. When interpreting results, it is essential to consider the geographic area of residence and the history of the suspected dog.

The performance of the EhrlichiaCHECK Ab ELISA was evaluated against the INgezim Ehrlichia ELISA, which uses the recombinant *E. canis* p30 protein [36]. The INgezim Ehrlichia ELISA showed 98.0% agreement with our ELISA and, when compared to IFAT, demonstrated sensitivity of 100% and specificity of 96.3%. In contrast, our ELISA, based on the *E. canis* gp19 antigen, exhibited both 100% sensitivity and 100% specificity. We identified one false-positive result with the INgezim Ehrlichia ELISA, which could be explained by the antigen used in the assay. In this context, we assessed potential cross-reactions through bioinformatics analysis of the p30 and gp19 proteins of *E. canis*. Based on the general rule according to which two sequences are considered homologous if they share more than 30% identity in length [47], we found that the p30 protein is similar to several other *Ehrlichia* spp. antigens, including the P44/Msp2 family outer membrane protein, surface antigen msp4, p28 antigen, OMP-1, p30-1, p30-2, and MAP1 proteins of *E. canis*, *E. minasensis*, *E. muris*, *E. japonica*, *E. chaffeensis*, *E. ruminantium*, and *E. ewingii*. In contrast, the *E. canis* gp19 antigen exhibited a narrower cross-reactivity spectrum, reacting only with other TRP19 homologous proteins from *E. minasensis*, *E. muris*, and *E. japonica*, as well as with the *E. muris* P13 ortholog protein [37, 58]. Additionally, TRP19 did not cross-react with antibodies in sera from dogs infected with *E. chaffeensis*, thereby providing species-specific discrimination between *E. canis* and *E. chaffeensis* infections [12]. This evidence may explain why the EhrlichiaCHECK Ab ELISA was more specific than the INgezim Ehrlichia ELISA.

Furthermore, false-negative results may occur in immunocompromised patients in which antibody concentrations below the lower detection limit of the assay could lead to negative results. Additionally, an animal may be serologically negative during the early stages of the disease or within the incubation period [4]. False-positives and false-negatives should be retested and, in any case, confirmed with another test. Diagnosing ehrlichiosis is complex, and the presence of antibodies to *E. canis* must be interpreted in conjunction with other diagnostic findings, such as disease progression, cross-reactivity with other *Ehrlichia* spp., and potential co-infections with other tick-borne diseases to reach a final diagnosis of CME.

Moreover, the earliest antibody response against *E. canis* is typically detected between 14 and 21 days post-infection, primarily targeting the gp36 and gp19 proteins [26, 39]. The gp19 protein is highly conserved among *E. canis* strains and can be detected in dog sera early during the acute phase of CME [39, 42]. As previously reported, ELISA tests based on the recombinant glycoproteins TRP36 (gp36) and TRP19 (gp19) have shown enhanced sensitivities compared to IFAT. In fact, antibodies against *E. canis* were detected on day 14 post-inoculation, up to 2 weeks earlier than with IFAT [12].

Stability tests were conducted at specific temperatures that reflect the climatic conditions of the regions where the product will be marketed [5, 40]. Once the product proves stable under storage conditions simulating a warmer climate, it is automatically considered suitable for use in colder areas. Accelerated stability studies are typically carried out at +37 °C, as this is the most effective method for speeding up the product’s degradation reactions, enabling a quick determination of its shelf life, rather than waiting for prolonged natural deterioration [10, 11]. Numerous accidental events, such as interruptions in the cold chain, may occur during shipping or storage and can negatively affect product’s quality and effectiveness. Unlike pharmaceutical products or veterinary drugs for which specific guidelines for stability testing are available [14, 24], there are no established regulations governing the shelf life of veterinary diagnostic kits. Stability studies conducted in accordance with the Standard Guide for Accelerated Aging of Sterile Medical Device Packages, ASTM F1980-21 [3] demonstrated that the kit remained stable for 6 weeks at +37 °C and for 18 months at the normal storage temperature of +2–8 °C.

## Conclusions

Since CME may be fatal if not treated and rapid diagnosis is needed, the new EhrlichiaCHECK Ab ELISA developed by Agrolabo S.p.A. is able to provide fast and accurate results about the presence or absence of anti-*E. canis* antibodies in approximately 35 min. It is sensitive and easy to perform, and may be more specific than other commercialized assays because it is based on a new antigen that limits cross-reactivities towards other *Ehrlichia* spp. In conclusion, the new kit may be a valuable tool for confirming exposure to *E. canis* in settings where more advanced techniques, such as IFAT, Western blotting, PCR, and tissue culture are not readily available.

## Data Availability

Data are available in this published article and as supplementary information. Datasets generated during the current study are also available from the corresponding author on reasonable request.

## Supplementary material

*Supplementary Table S1*. ELISA results after sample incubation time of 30 min. *Supplementary Table S2*. EhrlichiaCHECK Ab ELISA validation against IFAT. *Supplementary Table S3*. Analysis for cut-off determination. *Supplementary Table S4*. Detailed ELISA results obtained during reproducibility study. *Supplementary Table S5*. Comparison between EhrlichiaCHECK Ab ELISA (Agrolabo) and INgezim Ehrlichia ELISA (Gold Standard Diagnostics). *Supplementary Table S6*. Accelerated stability study. The supplementary material of this article is available at https://www.parasite-journal.org/10.1051/parasite/2025054/olm.
