# Supplementary material for: Evaluation of a newly developed rapid ELISA to detect anti-Ehrlichia canis antibodies in dogs
Source: Parasite. 2025 Sep 25;32:62. doi: 10.1051/parasite/2025054 (PMC12463349; doi:10.1051/parasite/2025054)
Supplement: Supplementary file 1 — Supplementary Table S1. ELISA results after sample incubation time of 30 min. [file parasite-32-62-s1.pdf]

Supplementary Table S1. ELISA results after sample incubation time of 30 minutes. Results are reported as OD values**.** Samples were tested on plates coated with 0.5 µg/mL or 1 µg/mL of *E. canis* gp19 antigen and incubated for 15 or 30 minutes. Cut-off: mean OD value of negative samples plus 3 times standard deviation, considering positive or negative samples with OD above or below 10% the cut-off, respectively. OD: optical density. POS: positive; NEG: negative.

Samples	IFAT	0.5 µg/mL (15 min)		0.5 µg/mL (30 min)		1 µg/mL (30 min)	
		OD	Results	OD	Results	OD	Results
Sample 1	NEG	0.060	NEG	0.068	NEG	0.068	NEG
Sample 2	NEG	0.073	NEG	0.073	NEG	0.075	NEG
Sample 3	NEG	0.275	NEG	0.236	NEG	0.239	NEG
Sample 4	NEG	0.047	NEG	0.048	NEG	0.048	NEG
Sample 5	POS	0.753	POS	0.772	POS	0.803	POS
Sample 6	POS	1.369	POS	1.372	POS	1.496	POS
Cut-off		0.437		0.367		0.372	
