# Supplementary material for: Evaluation of a newly developed rapid ELISA to detect anti-Ehrlichia canis antibodies in dogs
Source: Parasite. 2025 Sep 25;32:62. doi: 10.1051/parasite/2025054 (PMC12463349; doi:10.1051/parasite/2025054)
Supplement: Supplementary file 2 — Supplementary Table S2. EhrlichiaCHECK Ab ELISA validation against IFAT. [file parasite-32-62-s2.pdf]

Supplementary Table S2. EhrlichiaCHECK Ab ELISA validation against IFAT. Total number of samples analyzed: 112. IFAT kit: FLUO Ehrlichia canis (Agrolabo S.p.A.). Discordant samples are marked with an asterisk and highlighted in bold. OD: optical density. POS: positive; NEG: negative.

Samples	IFAT	EhrlichiaCHECK Ab ELISA (Agrolabo)	
		OD values	Results
1	NEG	0.141	NEG
2	NEG	0.066	NEG
3	NEG	0.080	NEG
4	NEG	0.062	NEG
5	NEG	0.057	NEG
6	NEG	0.076	NEG
7	NEG	0.079	NEG
8	NEG	0.060	NEG
9	NEG	0.073	NEG
10	NEG	0.065	NEG
11	NEG	0.055	NEG
12	NEG	0.048	NEG
13	POS	2.604	POS
14	POS	1.985	POS
15	POS	2.143	POS
16	POS	0.635	POS
17	POS	1.103	POS
18	POS	0.399	POS
19	NEG	0.054	NEG
20	POS	1.134	POS
21	NEG	0.275	NEG
22	NEG	0.124	NEG
23	NEG	0.148	NEG
24	NEG	0.106	NEG
25	NEG	0.047	NEG
26	NEG	0.054	NEG
27	NEG	0.060	NEG
28	NEG	0.066	NEG
29	POS	0.947	POS
30	NEG	0.125	NEG
31	POS	0.753	POS
32	POS	1.369	POS
33	NEG	0.166	NEG
34	NEG	0.146	NEG
35	POS	0.565	POS
36	POS	0.639	POS
37	NEG	0.145	NEG
38	NEG	0.063	NEG
39	NEG	0.093	NEG
40	POS	1.562	POS
41	POS	0.877	POS
42	NEG	0.061	NEG
43	NEG	0.069	NEG
44	POS	1.753	POS

Continued in the next page

Supplementary Table S2 *(continued).*

Samples	IFAT	EhrlichiaCHECK Ab ELISA (Agrolabo)	
		OD values	Results
45	NEG	0.074	NEG
46	NEG	0.076	NEG
47	NEG	0.073	NEG
48	POS	0.696	POS
49	POS	1.612	POS
50	POS	2.115	POS
51	POS	1.735	POS
52	POS	1.472	POS
53	POS	2.607	POS
54	POS	1.941	POS
55	POS	2.300	POS
56	POS	1.407	POS
57	POS	1.530	POS
58	POS	0.719	POS
59	POS	2.013	POS
60	POS	2.622	POS
61	POS	2.270	POS
62	POS	2.835	POS
63	POS	1.421	POS
64	POS	1.253	POS
65	POS	2.262	POS
66	POS	1.576	POS
67	POS	1.442	POS
68	NEG	0.052	NEG
69	NEG	0.057	NEG
70	NEG	0.056	NEG
71	NEG	0.049	NEG
72	NEG	0.052	NEG
73	NEG	0.050	NEG
74	NEG	0.093	NEG
75	POS	2.260	POS
76	NEG	0.068	NEG
77	POS	0.410	POS
78	POS	0.666	POS
79	POS	1.312	POS
80	NEG	0.167	NEG
81	NEG	0.056	NEG
82	NEG	0.058	NEG
83	POS	1.123	POS
84	NEG	0.067	NEG
85	NEG	0.108	NEG
86	NEG	0.061	NEG
87	NEG	0.077	NEG
88	POS	1.334	POS
89	NEG	0.116	NEG
90	NEG	0.053	NEG
91	NEG	0.084	NEG

Continued in the next page

Supplementary Table S2 *(continued).*

Samples	IFAT	EhrlichiaCHECK Ab ELISA (Agrolabo)	
		OD values	Results
92	POS	1.475	POS
93	POS	0.631	POS
94	POS	1.331	POS
95	NEG	0.083	NEG
96	NEG	0.090	NEG
97	POS	1.430	POS
98	NEG	0.073	NEG
99	NEG	0.066	NEG
100	POS	2.012	POS
101	NEG	0.082	NEG
102	NEG	0.095	NEG
103	POS	0.375	POS
**104***	**POS**	**0.157**	**NEG**
105	POS	1.359	POS
106	POS	1.745	POS
**107***	**NEG**	**1.526**	**POS**
**108***	**NEG**	**0.701**	**POS**
**109***	**NEG**	**1.558**	**POS**
110	NEG	0.055	NEG
111	NEG	0.058	NEG
**112***	**POS**	**0.051**	**NEG**
