# Supplementary material for: Evaluation of a newly developed rapid ELISA to detect anti-Ehrlichia canis antibodies in dogs
Source: Parasite. 2025 Sep 25;32:62. doi: 10.1051/parasite/2025054 (PMC12463349; doi:10.1051/parasite/2025054)
Supplement: Supplementary file 3 — Supplementary Table S3. Analysis for cut-off determination. [file parasite-32-62-s3.pdf]

Supplementary Table S3. Analysis for cut-off determination. Sp: Specificity; Se: Sensitivity; J: Jouden's index. PPV: Positive Predictive Value; NPV: Negative Predictive Value; LR+: positive likelihood ratio; LR-: negative likelihood ratio.

N. Cut-off	OD cut-off	Sp	1 - Sp	Se	Se-Sp	J	PPV	NPV	Accuracy	LR +	LR -	J max
1	0.030	0.000	1.000	1.000	1.000	0.000	0.455	-	0.455	1.000	-	0.912
2	0.040	0.000	1.000	1.000	1.000	0.000	0.455	-	0.455	1.000	-	
3	0.050	0.049	0.951	1.000	0.951	0.049	0.468	1.000	0.482	1.052	0.000	
4	0.060	0.279	0.721	0.980	0.702	0.259	0.532	0.944	0.598	1.359	0.070	
5	0.070	0.492	0.508	0.980	0.489	0.472	0.617	0.968	0.714	1.929	0.040	
6	0.080	0.623	0.377	0.980	0.357	0.603	0.685	0.974	0.786	2.600	0.031	
7	0.090	0.689	0.311	0.980	0.292	0.669	0.725	0.977	0.821	3.148	0.028	
8	0.100	0.754	0.246	0.980	0.226	0.734	0.769	0.979	0.857	3.987	0.026	
9	0.110	0.787	0.213	0.980	0.194	0.767	0.794	0.980	0.875	4.600	0.025	
10	0.120	0.803	0.197	0.980	0.177	0.784	0.806	0.980	0.884	4.984	0.024	
11	0.130	0.836	0.164	0.980	0.144	0.816	0.833	0.981	0.902	5.980	0.023	
12	0.140	0.836	0.164	0.980	0.144	0.816	0.833	0.981	0.902	5.980	0.023	
13	0.150	0.902	0.098	0.980	0.079	0.882	0.893	0.982	0.938	9.967	0.022	
14	0.160	0.902	0.098	0.961	0.059	0.862	0.891	0.965	0.929	9.768	0.043	
15	0.170	0.934	0.066	0.961	0.026	0.895	0.925	0.966	0.946	14.652	0.042	
16	0.180	0.934	0.066	0.961	0.026	0.895	0.925	0.966	0.946	14.652	0.042	
17	0.190	0.934	0.066	0.961	0.026	0.895	0.925	0.966	0.946	14.652	0.042	
18	0.200	0.934	0.066	0.961	0.026	0.895	0.925	0.966	0.946	14.652	0.042	
19	0.210	0.934	0.066	0.961	0.026	0.895	0.925	0.966	0.946	14.652	0.042	
20	0.220	0.934	0.066	0.961	0.026	0.895	0.925	0.966	0.946	14.652	0.042	
21	0.230	0.934	0.066	0.961	0.026	0.895	0.925	0.966	0.946	14.652	0.042	
22	0.240	0.934	0.066	0.961	0.026	0.895	0.925	0.966	0.946	14.652	0.042	
23	0.250	0.934	0.066	0.961	0.026	0.895	0.925	0.966	0.946	14.652	0.042	
24	0.260	0.934	0.066	0.961	0.026	0.895	0.925	0.966	0.946	14.652	0.042	
25	0.270	0.934	0.066	0.961	0.026	0.895	0.925	0.966	0.946	14.652	0.042	
26	0.280	0.951	0.049	0.961	0.010	0.912	0.942	0.967	0.955	19.536	0.041	
27	0.290	0.951	0.049	0.961	0.010	0.912	0.942	0.967	0.955	19.536	0.041	
28	0.300	0.951	0.049	0.961	0.010	0.912	0.942	0.967	0.955	19.536	0.041	
29	0.310	0.951	0.049	0.961	0.010	0.912	0.942	0.967	0.955	19.536	0.041	
30	0.320	0.951	0.049	0.961	0.010	0.912	0.942	0.967	0.955	19.536	0.041	
31	0.330	0.951	0.049	0.961	0.010	0.912	0.942	0.967	0.955	19.536	0.041	
32	0.340	0.951	0.049	0.961	0.010	0.912	0.942	0.967	0.955	19.536	0.041	
33	0.350	0.951	0.049	0.961	0.010	0.912	0.942	0.967	0.955	19.536	0.041	
34	0.360	0.951	0.049	0.961	0.010	0.912	0.942	0.967	0.955	19.536	0.041	
35	0.370	0.951	0.049	0.961	0.010	0.912	0.942	0.967	0.955	19.536	0.041	
36	0.380	0.951	0.049	0.941	-0.010	0.892	0.941	0.951	0.946	19.137	0.062	
37	0.390	0.951	0.049	0.941	-0.010	0.892	0.941	0.951	0.946	19.137	0.062	
38	0.400	0.951	0.049	0.922	-0.029	0.872	0.940	0.935	0.938	18.739	0.082	
39	0.410	0.951	0.049	0.902	-0.049	0.853	0.939	0.921	0.929	18.340	0.103	
40	0.420	0.951	0.049	0.902	-0.049	0.853	0.939	0.921	0.929	18.340	0.103	
41	0.430	0.951	0.049	0.902	-0.049	0.853	0.939	0.921	0.929	18.340	0.103	
42	0.440	0.951	0.049	0.902	-0.049	0.853	0.939	0.921	0.929	18.340	0.103	
43	0.450	0.951	0.049	0.902	-0.049	0.853	0.939	0.921	0.929	18.340	0.103	
44	0.460	0.951	0.049	0.902	-0.049	0.853	0.939	0.921	0.929	18.340	0.103	
45	0.470	0.951	0.049	0.902	-0.049	0.853	0.939	0.921	0.929	18.340	0.103	
46	0.480	0.951	0.049	0.902	-0.049	0.853	0.939	0.921	0.929	18.340	0.103	
47	0.490	0.951	0.049	0.902	-0.049	0.853	0.939	0.921	0.929	18.340	0.103	
48	0.500	0.951	0.049	0.902	-0.049	0.853	0.939	0.921	0.929	18.340	0.103	
49	0.510	0.951	0.049	0.902	-0.049	0.853	0.939	0.921	0.929	18.340	0.103	
50	0.520	0.951	0.049	0.902	-0.049	0.853	0.939	0.921	0.929	18.340	0.103	
51	0.530	0.951	0.049	0.902	-0.049	0.853	0.939	0.921	0.929	18.340	0.103	
52	0.540	0.951	0.049	0.902	-0.049	0.853	0.939	0.921	0.929	18.340	0.103	
53	0.550	0.951	0.049	0.902	-0.049	0.853	0.939	0.921	0.929	18.340	0.103	
54	0.560	0.951	0.049	0.902	-0.049	0.853	0.939	0.921	0.929	18.340	0.103	

Continued in the next page

Supplementary Table S3 *(continued).*

N. Cut-off	OD cut-off	Sp	1 - Sp	Se	Se-Sp	J	VPP	VPN	Accuracy	LR +	LR -
55	0.570	0.951	0.049	0.882	-0.068	0.833	0.938	0.906	0.920	17.941	0.124
56	0.580	0.951	0.049	0.882	-0.068	0.833	0.938	0.906	0.920	17.941	0.124
57	0.590	0.951	0.049	0.882	-0.068	0.833	0.938	0.906	0.920	17.941	0.124
58	0.600	0.951	0.049	0.882	-0.068	0.833	0.938	0.906	0.920	17.941	0.124
59	0.610	0.951	0.049	0.882	-0.068	0.833	0.938	0.906	0.920	17.941	0.124
60	0.620	0.951	0.049	0.882	-0.068	0.833	0.938	0.906	0.920	17.941	0.124
61	0.630	0.951	0.049	0.882	-0.068	0.833	0.938	0.906	0.920	17.941	0.124
62	0.640	0.951	0.049	0.824	-0.127	0.774	0.933	0.866	0.893	16.745	0.186
63	0.650	0.951	0.049	0.824	-0.127	0.774	0.933	0.866	0.893	16.745	0.186
64	0.660	0.951	0.049	0.824	-0.127	0.774	0.933	0.866	0.893	16.745	0.186
65	0.670	0.951	0.049	0.804	-0.147	0.755	0.932	0.853	0.884	16.346	0.206
66	0.680	0.951	0.049	0.804	-0.147	0.755	0.932	0.853	0.884	16.346	0.206
67	0.690	0.951	0.049	0.804	-0.147	0.755	0.932	0.853	0.884	16.346	0.206
68	0.700	0.951	0.049	0.784	-0.167	0.735	0.930	0.841	0.875	15.948	0.227
69	0.710	0.967	0.033	0.784	-0.183	0.752	0.952	0.843	0.884	23.922	0.223
70	0.720	0.967	0.033	0.765	-0.203	0.732	0.951	0.831	0.875	23.324	0.243
71	0.730	0.967	0.033	0.765	-0.203	0.732	0.951	0.831	0.875	23.324	0.243
72	0.740	0.967	0.033	0.765	-0.203	0.732	0.951	0.831	0.875	23.324	0.243
73	0.750	0.967	0.033	0.765	-0.203	0.732	0.951	0.831	0.875	23.324	0.243
74	0.760	0.967	0.033	0.745	-0.222	0.712	0.950	0.819	0.866	22.725	0.264
75	0.770	0.967	0.033	0.745	-0.222	0.712	0.950	0.819	0.866	22.725	0.264
76	0.780	0.967	0.033	0.745	-0.222	0.712	0.950	0.819	0.866	22.725	0.264
77	0.790	0.967	0.033	0.745	-0.222	0.712	0.950	0.819	0.866	22.725	0.264
78	0.800	0.967	0.033	0.745	-0.222	0.712	0.950	0.819	0.866	22.725	0.264
79	0.810	0.967	0.033	0.745	-0.222	0.712	0.950	0.819	0.866	22.725	0.264
80	0.820	0.967	0.033	0.745	-0.222	0.712	0.950	0.819	0.866	22.725	0.264
81	0.830	0.967	0.033	0.745	-0.222	0.712	0.950	0.819	0.866	22.725	0.264
82	0.840	0.967	0.033	0.745	-0.222	0.712	0.950	0.819	0.866	22.725	0.264
83	0.850	0.967	0.033	0.745	-0.222	0.712	0.950	0.819	0.866	22.725	0.264
84	0.860	0.967	0.033	0.745	-0.222	0.712	0.950	0.819	0.866	22.725	0.264
85	0.870	0.967	0.033	0.745	-0.222	0.712	0.950	0.819	0.866	22.725	0.264
86	0.880	0.967	0.033	0.725	-0.242	0.693	0.949	0.808	0.857	22.127	0.284
87	0.890	0.967	0.033	0.725	-0.242	0.693	0.949	0.808	0.857	22.127	0.284
88	0.900	0.967	0.033	0.725	-0.242	0.693	0.949	0.808	0.857	22.127	0.284
89	0.910	0.967	0.033	0.725	-0.242	0.693	0.949	0.808	0.857	22.127	0.284
90	0.920	0.967	0.033	0.725	-0.242	0.693	0.949	0.808	0.857	22.127	0.284
91	0.930	0.967	0.033	0.725	-0.242	0.693	0.949	0.808	0.857	22.127	0.284
92	0.940	0.967	0.033	0.725	-0.242	0.693	0.949	0.808	0.857	22.127	0.284
93	0.950	0.967	0.033	0.706	-0.261	0.673	0.947	0.797	0.848	21.529	0.304
94	0.960	0.967	0.033	0.706	-0.261	0.673	0.947	0.797	0.848	21.529	0.304
95	0.970	0.967	0.033	0.706	-0.261	0.673	0.947	0.797	0.848	21.529	0.304
96	0.980	0.967	0.033	0.706	-0.261	0.673	0.947	0.797	0.848	21.529	0.304
97	0.990	0.967	0.033	0.706	-0.261	0.673	0.947	0.797	0.848	21.529	0.304
98	1.000	0.967	0.033	0.706	-0.261	0.673	0.947	0.797	0.848	21.529	0.304
99	1.010	0.967	0.033	0.706	-0.261	0.673	0.947	0.797	0.848	21.529	0.304
100	1.020	0.967	0.033	0.706	-0.261	0.673	0.947	0.797	0.848	21.529	0.304
101	1.030	0.967	0.033	0.706	-0.261	0.673	0.947	0.797	0.848	21.529	0.304
102	1.040	0.967	0.033	0.706	-0.261	0.673	0.947	0.797	0.848	21.529	0.304
103	1.050	0.967	0.033	0.706	-0.261	0.673	0.947	0.797	0.848	21.529	0.304
104	1.060	0.967	0.033	0.706	-0.261	0.673	0.947	0.797	0.848	21.529	0.304
105	1.070	0.967	0.033	0.706	-0.261	0.673	0.947	0.797	0.848	21.529	0.304
