# Supplementary material for: Evaluation of a newly developed rapid ELISA to detect anti-Ehrlichia canis antibodies in dogs
Source: Parasite. 2025 Sep 25;32:62. doi: 10.1051/parasite/2025054 (PMC12463349; doi:10.1051/parasite/2025054)
Supplement: Supplementary file 4 — Supplementary Table S4. Detailed ELISA results obtained during reproducibility study. [file parasite-32-62-s4.pdf]

Supplementary Table S4. Detailed ELISA results during reproducibility study. ELISA tests were performed in 8 replicates on 3 plate batches (24 tests for each sample). PC: positive control; NC: negative control. Samples: negatives (n.1, n.2), positives (n.3, n.4).

Batches	N. tests	PC	NC	Sample 1 (-)	Sample 2 (-)	Sample 3 (+)	Sample 4 (+)
Batch 1	1	2.108	0.044	0.055	0.063	2.184	2.072
	2	2.198	0.046	0.057	0.066	2.136	2.093
	3	2.280	0.044	0.054	0.066	2.166	2.140
	4	2.207	0.044	0.057	0.068	2.259	2.175
	5	2.143	0.046	0.059	0.069	2.165	2.125
	6	2.155	0.049	0.055	0.067	2.256	2.165
	7	2.167	0.044	0.054	0.060	2.264	2.134
	8	2.233	0.048	0.061	0.062	2.260	2.202
Batch 2	1	1.983	0.047	0.054	0.062	2.031	2.076
	2	2.072	0.052	0.054	0.064	1.997	2.122
	3	2.011	0.050	0.056	0.061	1.930	2.087
	4	1.988	0.050	0.062	0.064	2.096	1.988
	5	2.010	0.055	0.054	0.057	2.065	2.076
	6	2.071	0.052	0.056	0.057	1.900	2.087
	7	2.057	0.047	0.052	0.057	1.994	2.126
	8	1.992	0.052	0.058	0.062	1.984	2.050
Batch 3	1	2.206	0.046	0.056	0.065	2.164	2.262
	2	2.143	0.044	0.062	0.062	1.934	2.106
	3	2.155	0.046	0.054	0.066	2.106	2.229
	4	1.993	0.047	0.054	0.056	2.090	2.003
	5	1.988	0.051	0.060	0.060	1.990	2.154
	6	2.005	0.053	0.054	0.062	2.096	2.171
	7	2.073	0.048	0.057	0.062	2.166	2.195
	8	2.110	0.047	0.054	0.064	2.119	2.166
