# Supplementary material for: Evaluation of a newly developed rapid ELISA to detect anti-Ehrlichia canis antibodies in dogs
Source: Parasite. 2025 Sep 25;32:62. doi: 10.1051/parasite/2025054 (PMC12463349; doi:10.1051/parasite/2025054)
Supplement: Supplementary file 5 — Supplementary Table S5. Comparison between EhrlichiaCHECK Ab ELISA (Agrolabo) and INgezim Ehrlichia ELISA (Gold Standard Diagnostics). [file parasite-32-62-s5.pdf]

Supplementary Table S5. Comparison results of EhrlichiaCHECK Ab ELISA (Agrolabo) and INgezim Ehrlichia ELISA (Gold Standard Diagnostics). Total number of samples analyzed: 51. Discordant samples are marked with an asterisk and highlighted in bold. OD: optical density. GSD: Gold Standard Diagnostics. POS: positive; NEG: negative.

Samples	IFAT	EhrlichiaCHECK Ab ELISA (Agrolabo)		INgezim Ehrlichia ELISA (GSD)	
		OD values	Result	OD values	Result
1	NEG	0.141	NEG	0.451	NEG
2	NEG	0.066	NEG	0.229	NEG
3	NEG	0.080	NEG	0.267	NEG
4	NEG	0.062	NEG	0.218	NEG
5	POS	2.604	POS	3.954	POS
6	POS	1.985	POS	1.667	POS
7	POS	2.143	POS	1.122	POS
8	POS	0.635	POS	1.540	POS
9	POS	0.947	POS	2.811	POS
10	NEG	0.125	NEG	0.185	NEG
**11***	NEG	0.146	NEG	**2.604**	**POS**
12	POS	0.565	POS	2.662	POS
13	POS	0.639	POS	2.431	POS
14	NEG	0.145	NEG	0.128	NEG
15	NEG	0.063	NEG	0.227	NEG
16	NEG	0.093	NEG	0.096	NEG
17	POS	1.562	POS	3.734	POS
18	POS	0.877	POS	3.893	POS
19	NEG	0.061	NEG	0.094	NEG
20	NEG	0.069	NEG	0.153	NEG
21	POS	1.753	POS	2.321	POS
22	NEG	0.074	NEG	0.173	NEG
23	POS	1.612	POS	2.444	POS
24	POS	2.115	POS	2.576	POS
25	POS	1.735	POS	1.632	POS
26	NEG	0.093	NEG	0.514	NEG
27	POS	2.260	POS	2.824	POS
28	NEG	0.068	NEG	0.178	NEG
29	POS	0.410	POS	2.755	POS
30	POS	0.666	POS	2.729	POS
31	POS	1.312	POS	2.547	POS
32	NEG	0.167	NEG	0.183	NEG
33	NEG	0.056	NEG	0.224	NEG
34	NEG	0.058	NEG	0.206	NEG
35	POS	1.123	POS	2.689	POS
36	NEG	0.067	NEG	0.521	DOUBTFUL
37	NEG	0.108	NEG	0.324	NEG
38	NEG	0.077	NEG	0.265	NEG
39	POS	1.334	POS	2.705	POS
40	NEG	0.116	NEG	0.528	DOUBTFUL
41	NEG	0.053	NEG	0.18	NEG
42	NEG	0.084	NEG	0.465	NEG
43	POS	1.475	POS	2.325	POS
44	POS	0.631	POS	2.744	POS

Continued in the next page

Supplementary Table S5 *(continued).*

Samples	IFAT	EhrlichiaCHECK Ab ELISA (Agrolabo)		INgezim Ehrlichia ELISA (GSD)	
		OD values	Result	OD values	Result
45	POS	1.331	POS	2.757	POS
46	NEG	0.083	NEG	0.538	DOUBTFUL
47	NEG	0.090	NEG	0.512	DOUBTFUL
48	POS	1.430	POS	2.746	POS
49	NEG	0.066	NEG	0.136	NEG
50	POS	2.012	POS	2.512	POS
51	NEG	0.082	NEG	0.141	NEG
