# Supplementary material for: Evaluation of a newly developed rapid ELISA to detect anti-Ehrlichia canis antibodies in dogs
Source: Parasite. 2025 Sep 25;32:62. doi: 10.1051/parasite/2025054 (PMC12463349; doi:10.1051/parasite/2025054)
Supplement: Supplementary file 6 — Supplementary Table S6. Accelerated stability study. [file parasite-32-62-s6.pdf]

Supplementary Table S6. Accelerated stability study. At each time of analysis ($T_0 – T_6$) samples were tested in duplicate and the mean OD values and percentage remaining activities (% RA) were calculated. PC: positive control; NC: negative control. Samples: negatives (1, 2); positives (3, 4).

Samples	T_0				T_2				T_4				T_6			
	OD 1	OD 2	Mean	% RA	OD 1	OD 2	Mean	% RA	OD 1	OD 2	Mean	% RA	OD 1	OD 2	Mean	% RA
PC	1.515	1.510	1.513	100	1.425	1.417	1.421	93.95	1.221	1.232	1.227	81.09	1.132	1.154	1.143	75.57
NC	0.042	0.043	0.043	100	0.041	0.043	0.042	98.82	0.041	0.040	0.041	95.29	0.042	0.044	0.043	101.18
Sample 1 (-)	0.052	0.052	0.052	100	0.054	0.052	0.053	101.92	0.051	0.052	0.052	99.04	0.048	0.048	0.048	92.31
Sample 2 (-)	0.055	0.057	0.056	100	0.061	0.060	0.061	108.04	0.055	0.058	0.057	100.89	0.055	0.053	0.054	96.43
Sample 3 (+)	1.901	1.909	1.905	100	1.901	1.900	1.901	99.76	1.786	1.788	1.787	93.81	1.538	1.597	1.568	82.28
Sample 4 (+)	1.662	1.667	1.665	100	1.505	1.501	1.503	90.30	1.420	1.426	1.423	85.49	1.212	1.259	1.236	74.23
